# Personal

**Published:** 1888-12

**Authors:** 


### Personal.

Dr. Charles A. L. Reed, of Cincinnati, has been chosen Dean of the Faculty of the Polyclinic, recently organized in that city. The Faculty is made up of prominent specialists, nearly all of whom are now, or have been, connected with one or the other of the three Cincinnati medical colleges. Dr. Reed himself was already Professor of Diseases of Women in two colleges, and this late honor adds another similar chair to his manifold duties. It is doubtful if a parallel exists in this country, *i. e.*, where one man holds three teaching chairs in the same department in his own city. At all events it is very unusual, and speaks volumes for the esteem in which the distinguished Cincinnatian is held by his professional confrerès.

Dr. N. S. Davis, editor of the *Journal of the American Medical Association*, has resigned, and Dr. John B. Hamilton, Supervising Surgeon-General United States Marine Hospital Service, has been appointed to fill the vacancy. This, we presume, accounts for Dr. Hamilton's recent resignation from the Marine Hospital Service, noted in the Associated Press dispatches a few days since. Dr. Hamilton possesses the qualifications as to age, experience, energy, and ability to make the *Journal* what it should be—a representative American medical weekly.

Dr. Joseph O'Dwyer, the originator of intubation of the larynx, and the inventor of the most approved instruments for the performance of the operation, has been appointed Professor of the Diseases of Children in the New York Post-Graduate Medical School and Hospital.

Ernest Wende, M. D., B. Sc., has been appointed Instructor in Botany in the Buffalo College of Pharmacy, to succeed Prof. Kellicott, who has accepted a professorship in the University of Ohio, at Columbus.—*American Druggist.*

Dr. Wm. H. Wathen, of Louisville, will read a paper on " Hysterectomy in Cancer of the Uterus," at the meeting of the Southern Surgical and Gynecological Association in the evening session, December 5th.

---

Dr. Edward Clark, Health Physician, attended the meeting of the American Public Health Association, in Milwaukee last month, as the official representative of the Board of Health of the City of Buffalo.

---

Dr. T. Gaillard Thomas, for many years one of the surgeons of the Woman's Hospital of the State of New York, has resigned. It is announced that Dr. Henry D. Nicholl has been appointed to fill the vacancy.

